# Accounting for effects of growth rate when measuring ecological stability in response to pulse perturbations

**DOI:** 10.1002/ece3.11637

**Published:** 2024-10-17

**Authors:** Andrea Mentges, Adam Thomas Clark, Shane A. Blowes, Charlotte Kunze, Helmut Hillebrand, Jonathan M. Chase

**Affiliations:** ^1^ German Centre for Integrative Biodiversity Research (iDiv) Halle‐Jena‐Leipzig Leipzig Germany; ^2^ Department of Computer Sciences Martin Luther University Halle Germany; ^3^ Institute of Biology, University of Graz Graz Austria; ^4^ Institute for Chemistry and Biology of Marine Environments [ICBM] Carl‐von‐Ossietzky University Oldenburg Wilhelmshaven Germany; ^5^ Helmholtz‐Institute for Functional Marine Biodiversity at the University of Oldenburg [HIFMB] Oldenburg Germany; ^6^ Alfred Wegener Institute, Helmholtz‐Centre for Polar and Marine Research [AWI] Bremerhaven Germany

**Keywords:** comparative analysis, evidence synthesis, growth rate dependence, meta‐analysis, stability measures

## Abstract

Ecological stability is a vital component of natural ecosystems that can inform effective conservation and ecosystem management. Furthermore, there is increasing interest in making comparisons of stability values across sites, systems and taxonomic groups, often using comparative synthetic approaches, such as meta‐analysis. However, these synthetic approaches often compare/contrast systems where measures of stability mean very different things to the taxa involved. Here, we present results from theoretical models and empirical data to illustrate how differences in growth rates among taxa influence four widely used metrics of ecological stability of species abundances responding to pulse perturbations: resilience, recovery, resistance and temporal stability. We refer to these classic growth‐rate‐dependent metrics as ‘realised’ stability. We show that realised resilience and realised temporal stability vary as a function of organisms' growth rates; realised recovery depends on the relation between growth rate and sampling duration; and realised resistance depends on the relation between growth rate and sampling interval. To account for these influences, we introduce metrics intended to be more independent of growth rates, which we refer to as ‘intrinsic’ stability. Intrinsic stability can be used to summarise the overall effects of a disturbance, separately from internal recovery processes – thereby allowing more general comparisons of disturbances across organisms and contexts. We argue that joint consideration of both realised and intrinsic stability is important for future comparative studies.

## Graphical abstract

We present results from theoretical models and empirical data to illustrate how differences in growth rates among taxa influence four widely used metrics of ecological stability of species abundances responding to pulse perturbations: resilience, recovery, resistance and temporal stability. To account for these influences on growth rate, we introduce ‘intrinsic’ stability metrics. Intrinsic stability can be used to summarise the overall effects of a disturbance, separately from internal recovery processes – thereby allowing more general comparisons of disturbances across organisms and contexts.

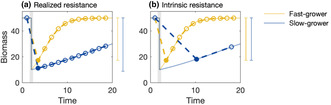

## 1 INTRODUCTION

Ecological stability relates to a family of concepts and associated metrics that describe how populations, communities or ecosystems respond to and recover from disturbances (Box 1). Understanding the factors that influence ecological stability can help to understand patterns of coexistence and biodiversity in natural communities (Loreau, 2010; May, 1973), and is of particular importance in light of the mounting effects of global change and disturbance driven by human activities (Čuhel et al., 2019; De Vries et al., 2012; Hautier et al., 2014; Hiddink et al., 2019; Li et al., 2020). However, stability is multi‐dimensional and can be quantified using many different metrics (Arnoldi et al., 2019; Donohue et al., 2013; Hillebrand et al., 2018; Pimm, 1984) (see Box 1), which can sometimes show contrasting trends (Domínguez‐García et al., 2019). Furthermore, even when the same metric is used, stability is often discussed in a comparative context, such as whether certain communities or ecosystems are more stable than others (De Vries & Shade, 2013; Lambert et al., 2014; Pimm, 1984; Tilman et al., 1996).

BOX 1 **Glossary of terms related to stability**

*System*: A group of organisms whose performance is of interest (e.g. a population, community or ecosystem).

*Disturbance*: An event that changes the function or composition of a system. We focus on disturbances that impact the state of the system (i.e. abundance), but not its underlying parameters (e.g. growth rates).

*Pulse disturbance*: A single, short‐term disturbance event (e.g. removal of 20% of the individuals or biomass in a plot).

*Environmental stochasticity*: Random fluctuations in environmental conditions that influence the function (e.g. fluctuations in temperature).

*Realised Resilience*: The speed at which the system returns to function as before a pulse disturbance. In this paper, we measure resilience as the per‐capita rate of recovery towards equilibrium after a disturbance, that is, (*x*(*t* + *ϵ*) – *x*(*t*))/*x̄*(*t*)/*ϵ*, where *x* describes the absolute differences between post‐disturbance abundance at time *t*, *N*_post_(*t*), and equilibrium abundance, *K*, such that *x*(*t*) = |*N*_post_(*t*) – *K*|; *ϵ* represents a small increment of time (e.g. one time step or sampling interval); and *x̄*(*t*) represents the average abundance between times *t* and *t* + *ϵ*. More generally (e.g. in multi‐species communities), we can also write these dynamics in terms of recovery towards an arbitrary equilibrium state N* (rather than *K*), such that d*N*/d*t* = *r g*(N*−*N*), given any monotonic decreasing function *g* for which *g*(N*−0) = 1 and *g*(0) = 0.

*Realised Resistance*: The impact of a disturbance on system state, which is jointly influenced by the attributes of the disturbance and the attributes of the organisms responding to the disturbance. This metric summarises the average displacement on the system by a particular kind of pulse perturbation (e.g. the impact of a flood or drought on biomass). Here, we measure resistance as the ratio between biomass measured immediately after a disturbance relative to biomass before the disturbance (i.e. *N*_dist_/*N*_pre_, where numbers closer to 1 indicate smaller impacts of the disturbance).

*Realised Recovery*: The degree to which the system re‐establishes its function after a pulse disturbance. In this paper, we measure recovery as the ratio of the state measured long after a disturbance has taken place relative to the pre‐disturbance state (i.e. *N*_end_/*N*_pre_, such that values close to 1 indicate complete recovery).

*Realised Temporal Stability*: The strength of fluctuations in function due to environmental stochasticity. Here, we define temporal stability as the inverse of the coefficient of variation (sometimes known as ‘invariability’) (i.e. mean(*N*)/std(*N*), such that larger values indicate less variability and thus greater stability).

While many comparative studies of ecological stability limit their scope to comparisons among a few types of species and ecosystems (Čuhel et al., 2019; De Vries & Shade, 2013; Hautier et al., 2014; Isbell et al., 2019; Knapp & Van Der Heijden, 2018), there is increasing interest in making comparisons across taxa and systems. For example, a number of studies in freshwater communities have imposed an experimental perturbation to a system (e.g. pesticides) and measured the temporal responses among different groups with very different life histories (e.g. plankton, plants and fish) (Polazzo et al., 2023; Polazzo & Rico, 2021). Similarly, long‐term observations of complex ecosystems in variable and disturbed environments often compare stability measures of organisms in different trophic levels (with very different life histories; Blüthgen et al., 2016). Likewise, as comparative and synthetic (e.g. meta‐analytic) approaches continue to gain in popularity, many studies explicitly compare stability across systems (Biggs et al., 2020; Hillebrand et al., 2018; Hillebrand & Kunze, 2020; Huang & Xia, 2019; Jones & Schmitz, 2009; Rip & McCann, 2011; Wisnoski et al., 2023; Xu et al., 2021). For example, variability tends to be lower and recovery is slower in terrestrial than in aquatic systems (Hillebrand & Kunze, 2020; Jones & Schmitz, 2009; Rip & McCann, 2011) and forested systems compared to grasslands and shrublands (Geng et al., 2019). However, several factors impede straightforward interpretation of these differences. First, stability depends on the nature of the underlying disturbance regime (e.g. acting on species' abundances or their vital rates), on whether disturbances act as ‘pulse’ (i.e. single disturbance events) or ‘press’ events (i.e. lasting changes to the ecosystem), as well as disturbance magnitude and frequency (Donohue et al., 2016; Radchuk et al., 2019). Second, the scale of sampling chosen for a particular study influences observed stability, such as the spatial grain (Wang et al., 2017), sampling duration (Bengtsson et al., 1997) and taxonomic resolution (Clark et al., 2021). Third, stability crucially depends on growth rates of species within the ecosystem (Bodmer et al., 1997), as well as correlates of growth rates, such as body size (Rip & McCann, 2011).

In this study, we explicitly address the effect of growth rates on stability, as this is critical for making comparisons of stability within and across ecosystem types. For simplicity and mathematical tractability, we will focus on systems undergoing pulse perturbations. These perturbations influence species' abundances but typically not their vital rates after the disturbance is relaxed (Arnoldi et al., 2019; Clark et al., 2021; Hillebrand & Kunze, 2020). We focus primarily on the dynamics of individual species, although many of our findings can be applied outside of these contexts, as discussed below (see Section 5.3).

### 1.1 Knowledge gap

Species can differ in growth rates by several orders of magnitude (e.g. from >5 day^−1^ for microbe communities to ~0.003 day^−1^ for tropical forest trees), and these differences strongly influence their measured stability (Degerman et al., 2013; Inman‐Narahari et al., 2014; Lankiewicz et al., 2016; Taylor et al., 2019). For example, fast‐growing, short‐lived species typically show higher recovery (Hillebrand et al., 2018; Lambert et al., 2014) and resilience (Capdevila et al., 2022; Čuhel et al., 2019; Hiddink et al., 2019; Hillebrand et al., 2018; Lobón‐Cerviá, 2009; McLaverty et al., 2020) than longer‐living, slower‐growing species; a forest may take centuries to recover from a disturbance, but microbes can recover within days. However, this raises the question: can one really conclude from these results that microbe populations are inherently more ‘stable’ than tree populations?

Some attempts have been made to address the impact of growth rate on realised measurements of stability. For example, recent theoretical studies of linear ecological models have revealed simple formulas describing the expected contribution of growth rate to stability metrics, such as resilience and the coefficient of variation (Arnoldi et al., 2016, 2019). Similarly, an empirical study by Hillebrand and Kunze (2020) accounted for some of the differences in longevity among taxonomic groups by normalising sampling time steps as a function of overall study duration, assuming that study durations were chosen to be proportional to the organism's lifespans. However, it is unclear how broadly these assumptions of linearity or study duration hold. Thus, more general approaches for formalising how different aspects of stability vary with growth rate, and how to account for these differences in comparative studies, are needed.

### 1.2 Aims of the study

Here, we discuss two classes of stability measures: ‘realised’ stability, which describes classic stability metrics (and that are therefore strongly influenced by growth rate); and a new class of metrics that we call ‘intrinsic’ stability (designed to be insensitive to underlying growth rates). While realised stability is jointly influenced by differences in growth rates and by other aspects such as disturbance regime or community structure, intrinsic stability is a specific result of biological and environmental factors *separate from* growth rate differences. Therefore, intrinsic stability may be useful for comparisons among sites, systems and organisms that differ greatly in their intrinsic growth rates and lifespans. Importantly, however, we do *not* advocate that intrinsic stability should replace realised stability as the only stability metric of interest. Rather, we suggest that both approaches can be applied in concert in order to explore different aspects of stability in ecological systems.

In what follows, we: (1) show how growth rate affects realised stability, using a simple conceptual model of a fast‐ and a slow‐growing species; (2) derive a series of intrinsic stability metrics, which summarise several growth‐rate‐independent aspects of ecological stability; and (3) apply our methods to an empirical dataset to demonstrate how realised and intrinsic stability can differ in real‐world contexts. We focus on four metrics (resilience, recovery, resistance and temporal stability – see definitions in Box 1), in relation to pulse perturbations that lead to changes in abundances, but not in underlying growth rates.

## 2 CONCEPTUAL ILLUSTRATION: HOW IS STABILITY AFFECTED BY GROWTH RATE?

**FIGURE 1:**
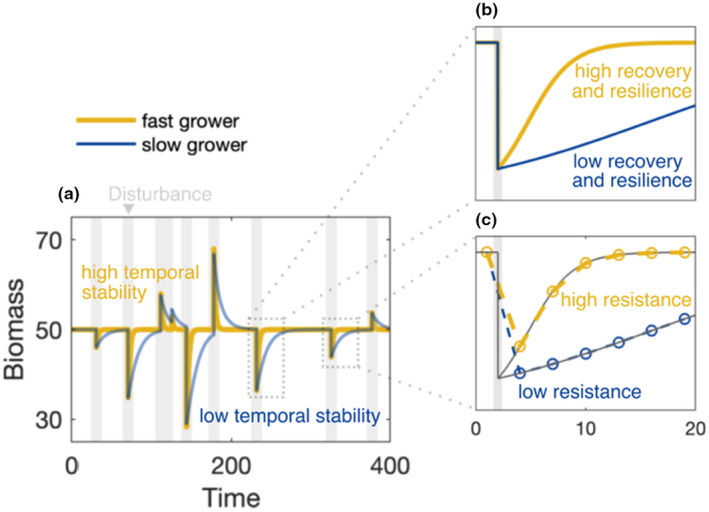
Realised stability depends on growth rate. Time series of biomass for two species undergoing logistic growth: a fast grower (yellow) and a slow grower (blue). Grey bars indicate a pulse disturbance (i.e. a sudden change in biomass) due to environmental stochasticity or external impact. Realised stability is illustrated in the form of (a) temporal stability, (b) recovery and resilience and (c) resistance based on discrete sampling points (circles). The fast grower is more stable compared to the slow grower for all four considered realised stability aspects.

To illustrate how ecological stability measurements are affected by growth rate, we simulated logistic growth of a ‘fast’‐ and a ‘slow’‐growing species, with growth rates *r*_fast_ = 0.5 and *r*_slow_ = 0.1, respectively, and carrying capacity *K* = 50. At the start of the simulation, the biomass of both species is in equilibrium (i.e. pre‐disturbance biomass equals carrying capacity *N*_pre_ = *K*). We then exposed the simulated species to one of two disturbance regimes. First, to test aspects of temporal stability, we simulated repeated stochastic pulse disturbance events (including both increasing and decreasing *N*) at random time points, representing environmental stochasticity (Figure 1a). Second, we simulate a single‐pulse disturbance by reducing the biomass *N*_pre_ by 40 (i.e. the disturbance strength is 80% of carrying capacity) at the time point *t*_dist_ = 4 (Figure 1b,c). In both cases, we simulated the system as a set of stochastic differential equations (Gillespie, 1976; Wilkinson, 2011). By simulating deterministic dynamics as continuous‐time processes and stochastic dynamics as discrete events, this approach allows for an ‘exact’ representation of individual trajectories taken by a stochastic dynamical system. In these simulations, we drew perturbation strength from a normal distribution with mean *μ* = 0, standard deviation *σ* = 10 and stochastic waiting times between disturbance events from an exponential distribution with mean frequency *λ* = 0.03. Between disturbance events, we solved the deterministic dynamics computationally (see below). We then observed the resulting dynamics using a fixed time step of 0.1 between measurements (note, this time window only impacts observations, not disturbance dynamics).

The slow‐ and fast‐growing species experienced the same stochastic disturbance regime. All realised stability (i.e. ‘classic’) metrics were calculated following the formulas in Box 1. Intrinsic metrics are described below. All simulations and analyses were performed using MATLAB R2021b and Statistics and Machine Learning Toolbox. Deterministic growth dynamics were simulated using the differential equation solver ode45 (based on existing code that had already been extensively de‐bugged for previous projects). All code and data for our analyses are available on GitHub (https://github.com/adamtclark/Mentges_growth‐rate‐stability), and is archived on Zenodo (https://zenodo.org/doi/10.5281/zenodo.11632219).

As expected, our simulations show that the fast‐growing species has higher realised temporal stability than the slow‐growing species in the face of repeated disturbances (Figure 1a), as well as higher realised recovery and resilience (Figure 1b). In theory, realised resistance is the same for both species (i.e. see grey solid lines in Figure 1c). However, because estimates of realised resistance are discrete in practice (i.e. depend on the time to the first post‐disturbance sampling event), a fast grower can recover to some degree before the first sample is taken and thus have higher realised resistance (see circles indicating slower sampling rates in Figure 1c).

## 3 PARTITIONING: HOW TO DERIVE INTRINSIC STABILITY FROM REALISED STABILITY

Next, we propose methods for deriving intrinsic stability from estimates of temporal stability, recovery, resilience and resistance that are less influenced by differences among species' growth rates. These represent, respectively, growth‐rate‐corrected estimates of fluctuations in the system in response to repeated pulse perturbations, the ability of the system to return to equilibrium, the rate at which recovery after disturbances occurs and impact of disturbances on biomass. These metrics can be calculated using four measurable aspects of the system: the time at which the last disturbance event took place *t*_dist_, the abundances observed before (*N*_pre_) and after (*N*_post_) the disturbance event and relative growth rate *r* (i.e. per‐capita growth rate achieved at low abundance).

While these metrics are not all formally independent of growth rate, they ensure that as long as species differ only in their intrinsic growth rates, measurements of their intrinsic stability will be similar. For example, in our conceptual illustration above, the slow grower and the fast grower have similar intrinsic stability despite differing substantially in realised stability (Appendix S1, Table S1).

### 3.1 Intrinsic temporal stability

**FIGURE 2:**
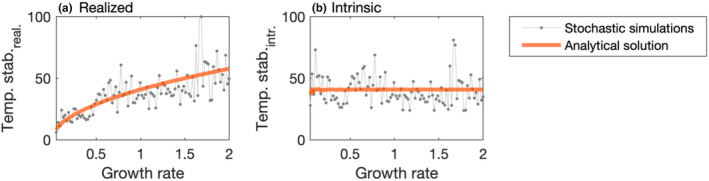
Partitioning of temporal stability estimates. (a) Realised temporal stability increases with growth rate, whereas (b) intrinsic temporal stability is on average constant across growth rates. Intrinsic temporal stability is derived by dividing realised invariability (i.e. 1/CV) by the square root of growth rate. The solid orange lines indicate the analytical relationships between temporal stability and growth rate (Equation 1) across a range of growth rates between 0.05 and 2. The dots indicate realised and intrinsic temporal stability of 100 independent stochastic time series, which deviate from the general trend due to the stochasticity in the time series.

We define temporal stability as the inverse of coefficient of variation (Box 1), and to control for growth rate, make use of the known relationship between the temporal variance of biomass *N* (i.e. the mean‐squared distance of the species' biomass from equilibrium) and properties of the disturbance regime for a univariate, linear system. This correction performs well even for more complex systems, such as logistic growth or Lotka–Volterra competition. Assuming that these properties of the disturbance regime (*μ*, *σ* and *λ*) are the same across time series, $\mathrm{var}\left(N\right)=\left(\mu^{2}+\sigma^{2}\right)\lambda /\left(2r\right)$ (Arnoldi et al., 2016, 2019; Clark et al., 2021), where *μ* is the average disturbance strength, *σ* is the standard deviation of environmental noise, *λ* is the frequency of the exponential distribution from which waiting times between disturbance events are drawn and *N* is biomass. Because $\mathrm{var}\left(N\right)={\mathrm{CV}}^{2}\text{mean}{\left(N\right)}^{2}$, it follows that $\mathrm{CV}\sqrt{r}=\sqrt{\left(\mu^{2}+\sigma^{2}\right)\lambda /2}/\text{mean}\left(N\right)=\text{const}$ for any given disturbance regime. That is, multiplying the raw observed realised CV by $\sqrt{r}$ yields a constant value, which summarises the effects of the disturbance regime on dynamics, but is independent of *r*. In terms of invariability (i.e. the inverse of CV), we define intrinsic temporal stability as (Figure 2):

(1) $$\text{temp}.\text{stab}._{\text{intr}}=\frac{1}{{\mathrm{CV}}_{\text{real}}\sqrt{r}}.$$

This metric can be calculated either at the scale of populations of a species (e.g. by fitting Equation (1) separately to the species‐level dynamics for each species in a community) or to an aggregate of many organisms or species (e.g. total community biomass dynamics).

### 3.2 Intrinsic recovery and resilience

For intrinsic recovery and resilience, differences among growth rates can be controlled for by re‐scaling the observed time axis. By stretching or shrinking the time axes in proportion to each species' growth rate, we can generate a shared time axis, where each unit of time represents the same amount of potential growth for each species. This is done by normalising the time axis such that all species grow with scaled growth rate *r* = 1. From the logistic growth function d*N*/d*t* = *rN* (*K*−*N*)/*K*, or general growth function d*N*/d*t* = *r g*(N*−*N*), the effect of growth rate is removed by dividing by *r*, resulting in a dimensionless time axis:

(2) $${\text{resilience}}_{\text{intr}}={\text{resilience}}_{\text{real}}/r.$$

If we define *τ* = *rt*, such that *t* = *τ*/*r*, we can re‐write Equation (2) as resilience_intr_ = d*N*/d*t* (1/*r*) = d*N*/d*t* d*t*/d*τ* = d*N*/d*τ*. Equation (2) thus shows that species will have similar realised resilience so long as they share similar rates of recovery relative to their own growth rate‐standardised time scale *τ*. In contrast, all else being equal, raw realised resilience will always tend to be faster for species with faster growth rates.

For intrinsic recovery, time axes are rescaled such that all species are able to achieve the same growth increment post‐disturbance. Specifically, we measure the rescaled time axis in units of time since disturbance (i.e. *t*_end_−*t*_dist_), where *t*_end_ is a single fixed time point that is maintained across all species in the sample. In practice, *t*_end_ can be fixed to any post‐disturbance time, although for ease of sampling it could be helpful to choose a biologically relevant time (e.g. the generation time for the slowest‐growing species in the sample). The reference time point at which recovery is measured for each species, *t*_intr_, is chosen such that *t*_intr_ = (*t*_end_−*t*_dist_) *r*, where *t*_end_ is time of sampling and *t*_dist_ is the time point of the disturbance, such that:

(3) $${\text{recovery}}_{\text{intr}}=N\left(\left(t_{\mathrm{end}}-t_{\text{dist}}\right)r\right)/N_{\mathrm{pre}}=N\left(t_{\text{intr}}\right)/N_{\mathrm{pre}}.$$

**FIGURE 3:**
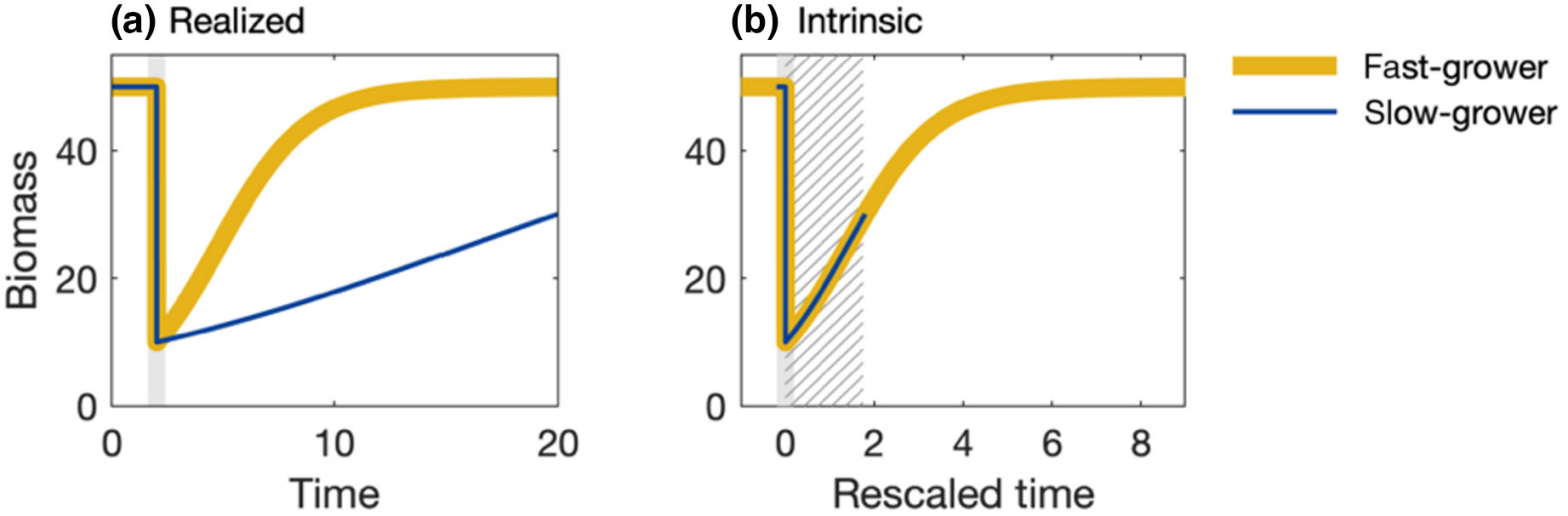
Comparison of realised versus intrinsic resilience and recovery estimates. Realised resilience and recovery are derived based on the (a) original time series. To derive intrinsic resilience and recovery, the original time series needs to be (b) re‐scaled. First, the time series is shifted so as the disturbance occurs at time equals zero. The rescaling illustrates that the time span sampled for the slow grower is effectively shorter. For the derivation of intrinsic resilience and recovery, only the time span shared by all species is used (hatched region).

Again, re‐defining time scales in terms of *τ* = *rt*, we find *t*_intr_ = (*τ*_end_/*r*−*τ*_dist_/*r*) *r* = *τ*_end_−*τ*_dist_. In words, *t*_intr_ represents the amount of time that has passed between the disturbance and the reference time point, rescaled to count in units of the growth rate‐standardised time scale *τ*. This rescaling ensures that the slower‐growing species is sampled longer than the faster‐growing species, so that the slow‐growing species is able to achieve the same degree of intrinsic recovery (Figure 3b). In contrast, when raw realised recovery is measured, the faster‐growing species is effectively given more time (relative to its growth rate) than the slower‐growing species to recover; especially if study durations are similar across species, which will lead to higher estimated realised recovery among faster‐growing species, regardless of the actual effects of the disturbance (Figure 3a).

### 3.3 Intrinsic resistance

**FIGURE 4:**
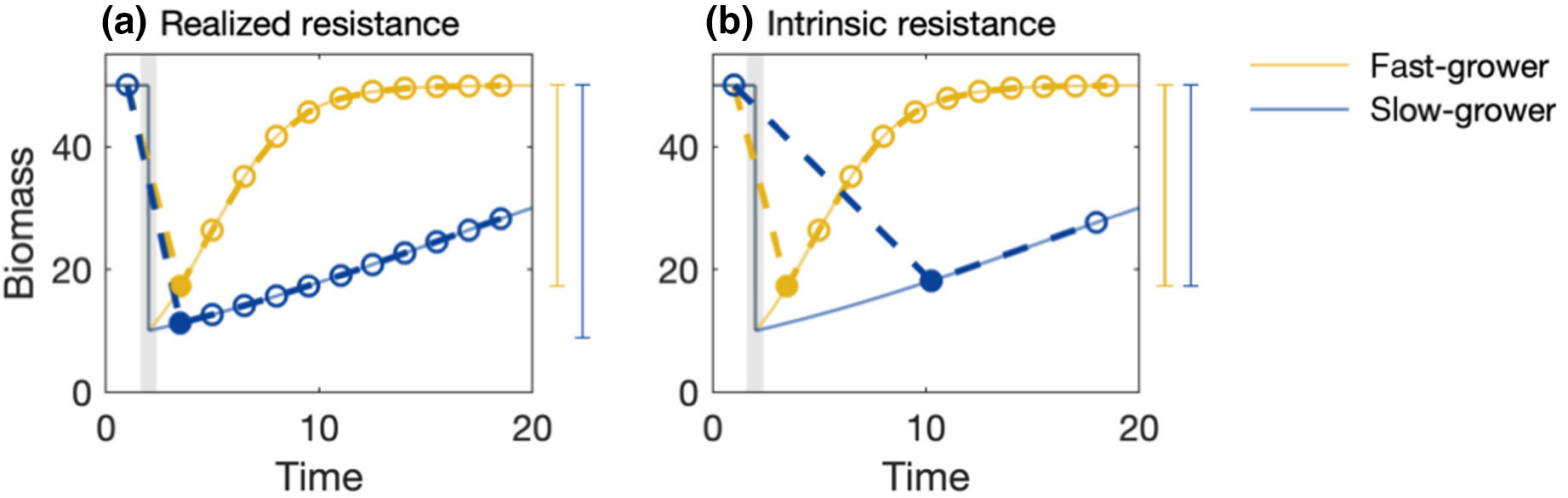
Partitioning resistance estimates. Realised resistance is calculated based on the amount of biomass lost at the (a) first time point after the disturbance (filled circle), which depends on the sampling interval. To derive intrinsic resistance, (b) the standard sampling interval is adapted to be proportional to growth rate. This is done by relating the growth rate of the slowest to the fastest‐growing species. After adapting the sampling interval, intrinsic resistance of the fast and slow grower is the same (see vertical bars to the left of each panel, indicating the inverse of resistance, i.e. the decrease in function due to the disturbance). The solid lines indicate the time series that would be observed for perfect sampling (i.e. very small sampling intervals).

For intrinsic resistance, re‐scaling the time axis alone is not sufficient because differences in measurements of realised resistance are introduced via finite sampling intervals. Resistance (in theory) jointly measures the overall intensity of the disturbance and the ability of the species to withstand that particular kind of disturbance. However, there will almost always be a lag between the moment when a disturbance occurs and when a sample is taken, leading to higher realised resistance among faster‐growing species that have had more of an opportunity to recover during the lag between disturbance and sampling (Figure 4a). To account for this effect in measurements of intrinsic resistance, we propose altering the reference time step chosen depending on each species' growth rate (Figure 4b). This correction is done by choosing a later time point for the intrinsic resistance calculation of the slow grower in order to give it time to ‘catch up’ with the faster grower. Although both intrinsic resistance estimates for the fast‐ and slow‐growing species will be biased – since they do not perfectly capture the response immediately after the disturbance – they will be comparable in terms of the overall opportunity that each species is given to recover from the disturbance.

To describe how the correction of time step should look, let us assume that a fast‐ and slow‐growing species are both initially impacted equally by a perturbation (i.e. both their biomasses are reduced to the same fraction of their carrying capacity). Our goal is to find some extended post‐disturbance observation time for the slow‐growing species such that it has had the same opportunity to recover as the fast‐growing species (i.e. d*N*_fast_ = d*N*_slow_)_._ For logistic growth, the change in biomass following the disturbance is described for the fast grower as d*N*_fast_/d*t*_fast_ = *r*_fast_ *N*_fast_ (*K*−*N*_fast_)/*K* and, for the slow grower as d*N*_slow_/d*t*_slow_ = *r*_slow_ *N*_slow_ (*K*−*N*_slow_)/*K*, scaling biomasses for each species such that their *K* values are equal. For this logistic system, the time span needed to achieve d*N*_fast_ = d*N*_slow_ is d*t*_slow_ = (*r*_fast_ *N*_fast_ (*K*−*N*_fast_)/*K*)/(*r*_slow_ *N*_slow_ (*K*−*N*_slow_)/*K*) d*t*_fast_. Since both initial impact of the perturbation and recovery post‐perturbation are (by definition) identical between the species, we know that *N*_fast_ = *N*_slow_. Thus, d*t*_slow_ = *r*_fast_/*r*_slow_ d*t*_fast_, and intrinsic resistance can be measured as:

(4) $${\text{resistance}}_{\text{intr}}=N\left(t_{\text{dist}}+r_{\text{fast}}/r_{\text{slow}}{dt}_{\text{fast}}\right)/N_{\mathrm{pre}}=N\left(t_{\text{dist}}+{dt}_{\text{slow}}\right)/N_{\mathrm{pre}}.$$

More generally, d*t*_slow_ can also be found via simulation of more complex models, for example, by identifying a time interval d*t*_slow_ for which the recovery of the slower‐growing species is expected to be equal to the recovery experienced by the fast‐growing species after time interval d*t*_fast_. When measured across many different organisms or species, d*t*_fast_ is defined as the time increment between the disturbance and the first post‐disturbance observation for the fastest grower, *r*_fast_ is defined as the growth rate for that fastest grower and *r*_slow_ is the growth rate for each of the other remaining species or organisms. Note that in some real‐world time series, it may not be possible to find a sampling event that corresponds perfectly to *t*_dist_ + d*t*_slow_. Under these circumstances, abundance at this time point would need to be approximated, from simulations or interpolation from other nearby time steps.

## 4 ILLUSTRATION: INTRINSIC AND REALISED STABILITY IN EMPIRICAL DATA

To illustrate how large an impact growth rate differences among organisms can have on stability estimates, and how intrinsic stability can be calculated in practice, we apply the methods described above to a published dataset by Hillebrand and Kunze (2020). We chose this dataset because it is open access, contains a wide range of taxonomic groups from both aquatic and terrestrial ecosystems and includes estimates of the relative growth rate for each species group from the published literature (Appendix S3; Ahn et al., 2013; Benke & Jacobi, 1986; Calbet & Landry, 2004; Houghton et al., 2013; Lankiewicz et al., 2016; Levang‐Brilz & Biondini, 2003; Liang & Uye, 1997; McConville et al., 2016; Møhlenberg, 1995; Nielsen & Sand‐Jensen, 1991; Piwosz et al., 2018; Ramírez & Pringle, 2006; Reddy & Debusk, 1984; Schmidt et al., 2019). Nevertheless, a major caveat with these analyses is that the growth rate estimates taken from group‐level averages across many species, and were not measured under the same context as the disturbance data themselves, they include significant errors. Thus, while the *methods* and *qualitative results* that we present here provide a useful guide and motivation for future studies, our specific *quantitative estimates* should not necessarily be taken as accurate estimates of intrinsic stability for these species groups. As discussed below, we strongly urge future stability studies to measure growth rates in situ as part of their study design, as they will yield much more accurate and reliable estimates of intrinsic stability.

The dataset is highly heterogeneous, comprising samples from a wide geographic range, including open and closed systems, field and mesocosm studies and a variety of disturbance types. We focus only on controlling for the variability introduced through growth rate differences, while the other heterogeneities influencing stability remain (e.g. differences in disturbance regime, community composition or environmental conditions). Information on the studied organisms, habitats and other features for each observation was taken from Appendix S1 of the original study (Hillebrand & Kunze, 2020). Each individual study included both ‘control’ (i.e. undisturbed) and ‘treatment’ (i.e. disturbed) replicates. In lieu of information on carrying capacity, we took the log‐response ratios of treatments versus controls reported in the original study and exponentiated them to convert them to treatment/control ratios (i.e. *N*_treat_(*t*)/*N*_contr_(*t*)). The log‐response ratios of treatment to control are available online at https://datadryad.org/stash/dataset/doi:10.5061/dryad.cz8w9gj09. This approach assumes that the control state is representative of the local equilibrium, and therefore that the treatment state shows deviations from that equilibrium. Thus, we assume that treatment/control ratios near 1 are indicative of recovery towards equilibrium, whereas ratios far from 1 indicate lingering effects of the disturbance. We therefore follow the same formula for realised stability as in Box 1, with *N*_pre_ = *K* = 1 and *N*_post_ = *N*_treat_/*N*_contr_.

### 4.1 Data cleaning

We restricted the dataset to data reflective of species abundances (i.e. biomass, number of individuals or cover). For both vertebrates (sub‐classified to fish and birds) and plants (sub‐classified to forbs, grasses, marsh plants, algae, woody plants and mixed), the groupings were too broad to assign a common growth rate to the whole group, and thus we sub‐classified them using information from the original studies. We excluded all species groups with less than 10 observations in the dataset (see Appendix S3). We also excluded the category ‘mixed plants’ as both woody and non‐woody plants were included in the same study (that differ substantially in growth rates). Our restricted dataset included seven species groups: microbes, phytoplankton, periphyton, macroinvertebrates, zooplankton, grasses and macrophytes. We identified three observations with unusually high resistance (>50 times greater than the median) as outliers which we excluded from analyses (however, including them did not change the general pattern) (case ID ‘CK036_1’; (Battaglia et al., 2005), case ID ‘CK030_9’; (Downing et al., 2008) and case ID ‘HH024_3’; (Benstead et al., 2007)).

### 4.2 Growth rates of species groups

We compiled estimates of growth rates for the seven species groups from the published literature. We searched for published estimates of relative growth rate (sometimes called ‘instantaneous growth rate’) from studies following the definition of Hunt and Cornelissen (1997) and Lugert et al. (2016), with the formula RGR = (ln(*w*_*t*_) *–* ln(*w*_*i*_))/(Δ*t*), where *w*_*t*_ is biomass at the end of the sampling, *w*_*i*_ is initial biomass and Δ*t* is the sampling duration. For each species group, we derived at least three independent estimates from at least two different studies. For each species group, we calculated the mean of the collected estimates and used this for subsequent analyses (Appendix S3).

#### 4.2.1 Realised stability in the empirical data

For each of the seven species groups (microbes, phytoplankton, periphyton, macroinvertebrates, zooplankton, grasses and macrophytes), we calculated realised stability measures based on the time series of treatment/control ratios (see Box 1). The mean estimate of relative growth rate derived from the literature ranged from 0.08 day^−1^ for macrophytes to 1.38 day^−1^ for microbes.

**FIGURE 5:**
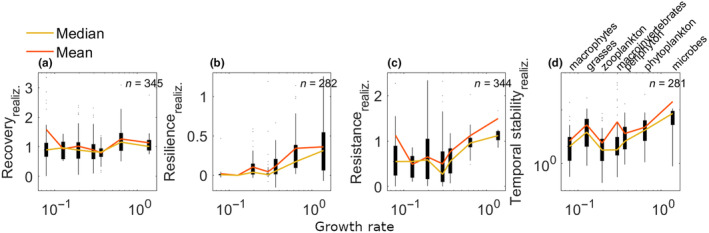
Realised stability as a function of growth rates in an empirical dataset (Hillebrand & Kunze, 2020). Horizontal access shows growth rates of seven species groups (in units of 1/day) plotted against stability measures of (a) recovery, (b) resilience, (c) resistance and (d) temporal stability (on logarithmic scale). These measures of realised stability do not account for differences in growth rates across groups. Variability of stability measures is visualised as a boxplot, where the edges of the black boxes indicate the 25th and 75th percentiles. The coloured lines indicate the median (yellow) and mean (orange) stability per species group. We report here the stability measures that we calculated as specified in the methods and Box 1.

Interestingly, we found that recovery did not change systematically with growth rate (Figure 5a), potentially indicating that empiricists had indeed chosen biologically comparable time windows for the species groups in the studies. In contrast, resilience, resistance and temporal stability tended to increase with growth rate (Figure 5b–d).

#### 4.2.2 Intrinsic stability in the empirical data

**FIGURE 6:**
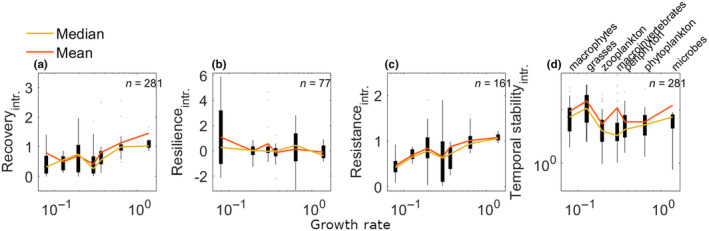
Intrinsic stability as a function of growth rate among empirical datasets (Hillebrand & Kunze, 2020). Horizontal axis shows growth rates (1 day^−1^) plotted against intrinsic stability metrics that are meant to account for the influence of growth rate on (a) intrinsic recovery, (b) intrinsic resilience, (c) intrinsic resistance and (d) intrinsic temporal stability (on logarithmic scale). Variability of stability measures is visualised as a boxplot, where the edges of the black boxes indicate the 25th and 75th percentiles. The coloured lines indicate the median (yellow) and mean (orange) stability per species group.

Next, we derived estimates of intrinsic stability for each species group using the methods described above. Again, we stress that: (1) we do not intend for these intrinsic stability measures to replace realised stability metrics, rather that they have complementary ecological interpretations; and (2) due to error in the growth rate estimates used here, quantitative comparisons among groups would be unwise. Nevertheless, these intrinsic estimates provide an additional tool with which to compare and contrast disturbance effects across different contexts and demonstrate the large effects that differences in growth rates can have on realised stability measurements (Figure 6).

In general, we found that realised versus intrinsic stability differed both in magnitude and in their relation to relative growth rate. On average, intrinsic recovery increased with growth rate (whereas realised recovery was relatively unchanged); in contrast, intrinsic temporal stability and intrinsic resilience did not show any clear trends (whereas realised temporal stability and realised resilience generally increased with growth rate). Resistance was the only aspect of stability that showed a similar trend for both intrinsic and realised metrics, with both increasing as a function of growth rate.

## 5 DISCUSSION

### 5.1 General theoretical findings

Our basic theoretical model and illustrative empirical dataset demonstrate that all four realised stability measures – recovery, resilience, resistance and temporal stability – vary strongly as a function of the growth rates of the species being studied. Indeed, growth rates have been empirically linked to recovery (Hillebrand et al., 2018; Lambert et al., 2014) and resilience (Čuhel et al., 2019; Hiddink et al., 2019; Hillebrand et al., 2018; Lobón‐Cerviá, 2009). Empirical evidence for resistance is more mixed, where some studies report that faster‐growing species are more resistant to harvesting (Bodmer et al., 1997; Hiddink et al., 2019), whereas others suggest that resistance is lower in short‐lived, fast‐growing species due to a trade‐off between resilience and resistance (De Vries et al., 2012; De Vries & Shade, 2013). Such a negative correlation between resilience and resistance has been reported several times (Hillebrand et al., 2018; Li et al., 2020; Orwin et al., 2006) and could be explained by differences in species' metabolic strategies (Li et al., 2020). In contrast, we observe a positive correlation between resilience and resistance. Nevertheless, for systems where sampling rates are slow relative to system dynamics, it is clear that lags between disturbance events and measurements can also lead to biases in measurements of resistance, which grow stronger as a function of growth rate (Clark et al., 2021).

### 5.2 Trends of realised stability with growth rate in the empirical data

Possibly our most important result is that the direction of trends in the empirical data changed for intrinsic versus realised stability (i.e. after accounting for differences in growth rate). These large differences – driven by biologically realistic variation in intrinsic growth rate among species groups – are our primary justification for advocating that both realised and intrinsic stability should be analysed in concert. Interestingly, despite the high levels of noise likely present in our growth rate estimates, realised resilience, resistance and temporal stability all increase with growth rate in the empirical dataset, roughly in accordance with biological expectations. Given the strong link between growth and recovery rates, it is not particularly surprising that we find such a strong link between resilience and growth rate. Note, however, that the two rates are distinct, as growth rates track the per‐capita rate of change in abundance (i.e. d*N*/d*t*/*N*), whereas resilience tracks the rate at which abundances return towards equilibrium (i.e. d(*N*−*K*)/d*t*/(*N*−*K*)). The increase in realised resistance with growth rate is expected because of sampling rate; relative to their intrinsic growth rates, fast growers are typically studied using a slower sampling interval than slow growers, giving the faster growers more time to recover before the first sampling point, which results in higher observed resistance. Fast‐growing organisms are thus not necessarily more ‘stable’ than slow‐growing organisms.

One exception was realised recovery, which did not behave as we expected, but instead was largely independent of growth rate. One possible explanation for this is that the authors of the original studies generally allowed near full recovery following disturbance, regardless of growth rate (i.e. longer experiments for longer‐lived organisms; Hillebrand & Kunze, 2020). Such a priori adaptation of the sampling duration effectively removes the effect of growth rate from recovery estimates. Alternatively, this result may simply be indicative of excess noise in our growth rate estimates, which may have obscured any other underlying patterns. Again, in order to facilitate more accurate estimates of intrinsic stability, we advocate that future stability studies quantify organisms' intrinsic growth rates in situ as part of their study design. These in situ growth rates can be derived from many different sources, such as lab or monoculture experiments, fitting time‐series data to population growth models or even based on morphometric measurements or associations with functional traits.

One other result that was general across our empirical analyses is that even after accounting for effects of growth rate on stability measurements, there was still substantial unexplained variability in intrinsic stability both across and within species groups. While some of this variability is doubtlessly a result of our noisy growth rate estimates, it is also demonstrative of the fact that many other factors can have a large influence on measures of stability (e.g. disturbance type, the size and composition of the species pool and sampling effort). Indeed, stability measures are highly context dependent (Radchuk et al., 2019). Thus, by analysing how both realised and intrinsic stability varies across sites, systems and organisms, it should be possible to separately characterise the contributions of growth rate, versus these other factors, to system stability. As an example, see Figure S1 in Appendix S2, in which we show a theoretical case where differences in carrying capacity (*K*) influence stability measures (Appendix S2). For two species with similar growth rates, the high‐carrying capacity species shows higher stability in all four metrics because the disturbance is relatively less severe. Measuring intrinsic and realised stability thus represents a useful tool to disentangle the effect of abiotic and biotic factors on overall system stability.

Our results also echo the findings of other studies showing that stability is a multidimensional concept and there is not one stability metric which in isolation describes a systems dynamic following perturbation (Domínguez‐García et al., 2019; Donohue et al., 2013; Hillebrand et al., 2018; Hillebrand & Kunze, 2020). Instead, different aspects of stability can show different patterns: for example, while resilience and resistance tend to be negatively correlated with each other but positively with recovery, respectively, temporal stability is less correlated with other metrics. Consequently, a more holistic assessment of change and stability is required to accurately assess systems dynamics following perturbation.

### 5.3 Applications in multi‐species systems

We anticipate that intrinsic stability will be calculated on a species‐by‐species basis, or for some aggregation of species, such as total community biomass. In general, however, these approaches should be valid even in relatively complex, diverse communities. For communities with simple linear dynamics, the derivations for temporal stability, resilience and recovery should all hold, while the formula for resistance also holds for systems undergoing logistic growth. However, most of the metrics discussed above are still effective in summarising system behaviour, such as multi‐species Lotka‐Volterra models (Arnoldi et al., 2019; Barbier et al., 2018), or models with spatially or temporally structured environments (Clark et al., 2021). That said, certain types of more complex models, such as complex food webs (Neutel et al., 2002) or models that track the flow of energy and matter (Bastolla et al., 2005; Rip & McCann, 2011), could lead to behaviour that our metrics have difficulty tracking. This is especially true if they drive populations far away from their equilibrium states, or lead to complex transient or non‐stationary behaviour (Chesson, 2017).

For these more complex cases, we can offer three possible solutions. First, for a broad class of systems, complex interactions among many species can lead to relatively simple system‐level behaviour, which can be predicted from relatively basic assumptions drawn from statistical mechanics and disordered systems theory (Barbier et al., 2018; Stone, 2016). For these cases, we expect our methods to work well as described above, with very little adaptation needed. Second, if an explicit, already parameterised model for the system already exists, then each of the metrics can be derived in terms of the simulated dynamics of the model (i.e. *N*(*t*)) relative to the expected equilibrium (N*) and estimated relative growth rate (i.e. growth rate at near‐zero abundance). Similar methods have been used to study coexistence in complex systems (Ellner et al., 2019). Finally, even without a priori information about the underlying equations governing system behaviour, non‐parametric forecasting methods can be applied to estimate transient trajectories and associated stability properties with relatively few assumptions (Clark et al., 2022).

## 6 CONCLUSIONS

This paper shows how classic ‘realised’ measurements of ecological stability can be strongly influenced by differences in organisms' intrinsic growth rates, and demonstrates how to correct for these effects to generate estimates of ‘intrinsic’ stability. Importantly, we show that the effect of growth rate differences on realised stability can be quite high, even when sampling is standardised (e.g. the same time span sampled), regardless of the metric used (i.e. temporal stability, resilience, recovery and resistance are all affected by growth rate), or the ecological scale being studied (i.e. individuals, populations or communities).

Overall, these findings highlight the critical role that temporal scale dependence, in addition to spatial scale dependence (Wang et al., 2017), plays in stability measures, particularly when comparing across sites, systems and organisms. Future comparative studies should more specifically acknowledge, and ideally incorporate, the effect of growth rate on estimates of ecological stability. To do so, we suggest that studies should strive to measure in situ relative growth rates, and explicitly report both realised and intrinsic measures of stability. These in situ growth rates can be derived from many different sources, such as lab or monoculture experiments, parameterising population growth models using time‐series data, or even based on morphometric measurements or associations with functional traits. The resulting contrasts between these realised and intrinsic metrics could help identify more general patterns in how different organisms respond to specific disturbance types – and, thus, help design strategies for better predicting and mitigating these effects in the future.

## AUTHOR CONTRIBUTIONS

**Andrea Mentges:** Conceptualization (equal); writing – original draft (equal). **Adam Thomas Clark:** Conceptualization (equal); writing – review and editing (lead). **Shane A. Blowes:** Conceptualization (equal). **Charlotte Kunze:** Writing – review and editing (supporting). **Helmut Hillebrand:** Conceptualization (equal); writing – review and editing (supporting). **Jonathan M. Chase:** Conceptualization (equal); writing – review and editing (lead).

## CONFLICT OF INTEREST STATEMENT

The authors declare no competing interests.

## Supporting information


Appendix S1

## Data Availability

All code and data for our analyses are available on Github at https://github.com/adamtclark/Mentges_growth‐rate‐stability, and is permanently archived on Zenodo at: http://zenodo.org/records/11632220.
