# Supplementary material for: Accounting for effects of growth rate when measuring ecological stability in response to pulse perturbations
Source: Ecol Evol. 2024 Oct 17;14(10):e11637. doi: 10.1002/ece3.11637 (PMC11483556; doi:10.1002/ece3.11637)
Supplement: Supplementary file 1 — Appendix S1 [file ECE3-14-e11637-s001.docx]

# Appendices

#### **Appendix 1**

**Table S1: Stability for a slow-grower (r=0.1) and a fast-grower (r=0.5), corresponding to the simulations in Figure 1.** The realised stability is based on standard measures of stability, where high values indicate high stability. The intrinsic stability refers to estimates which account for the influence of growth rate by applying our proposed methods, with a more specific derivation described below. Bold printed numbers indicate substantial differences in stability between the two species.

|  | Realised stability | |  | Intrinsic stability | |
| --- | --- | --- | --- | --- | --- |
|  | *Slow-grower* | *Fast-grower* |  | *Slow-grower* | *Fast-grower* |
| Temporal stability | 11.57 | **24.61** |  | 36.57 | 34.80 |
| Recovery | 0.60 | **1.00** |  | 0.60 | 0.60 |
| Resilience | 0.06 | **0.50** |  | 0.60 | 0.60 |
| Resistance | 0.23 | **0.40** |  | 0.40 | 0.40 |

#### **Appendix 2**

### Conceptual illustration: Influence of carrying capacity on stability

Similar to growth rate, stability is also affected by the carrying capacity. Here, we illustrate this by showing two species with the same growth rate, but different carrying capacity. This scenario could represent for example two species of the same species growing in a “good” (*K_high_* = 80) and a “bad” (*K_low_* = 50) environment. Both species experience a disturbance of the same absolute strength (i.e. biomass is reduced by the same amount in the two cases). Stability is measured by the same metrics as in the conceptual simulation on the influence of growth rate (see main text).

The high-carrying capacity species shows higher stability in all four metrics. It recovers faster, more fully and is more resistant, because the disturbance is relatively seen less severe, removing only about half the biomass (versus 80% in the low carrying capacity case). The high-capacity species is also more temporally stable because, while the standard deviation of its biomass is the same as in the bad environment, the mean is higher (*temp. stab. =* *mean*(*N*)*/std*(*N*)).


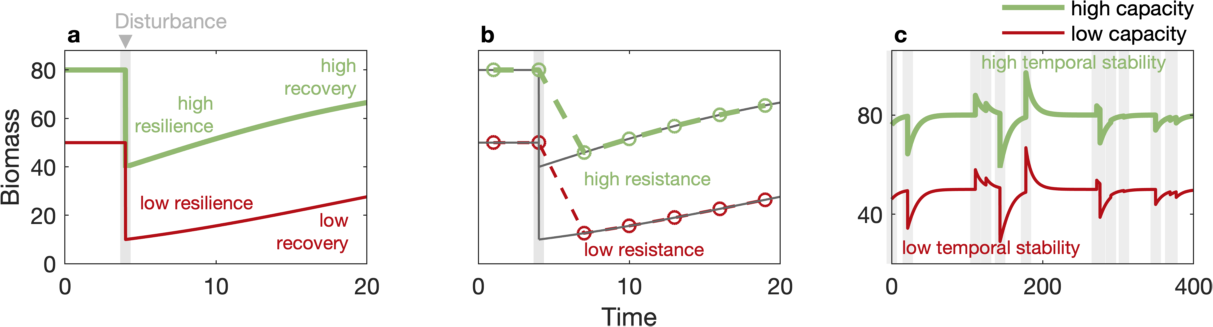


**Figure S1: Effect of carrying capacity on stability estimates.** Time series of biomass *N* for two species showing logistic growth, one with a low carrying capacity (red, *K* = 50) and one with a high carrying capacity (light green, *K* = 80). Grey bars indicate a disturbance, i.e. a sudden change in biomass. Stability is illustrated in the form of (**a**) resilience and recovery, (**b**) resistance, based on discrete sampling points, and (**c**) temporal stability in the face of a stochastic disturbance regime over a longer time period. For the species with high carrying capacity, stability to these disturbances is higher for all four considered stability aspects (see Table S2).

**Table S2: Stability for a species with low carrying capacity (*K*=50) and one with high capacity (*K*=80), corresponding to Figure S1**. Realised stability is derived using simple measures of stability, where high values indicate high stability: resilience = maximum linear slope in biomass between two sampling points, recovery = ratio of biomass at the end of the simulation to carrying capacity, resistance = ratio of biomass at the first sampling point after the disturbance to carrying capacity, and temporal stability = inverse coefficient of variation.

|  | Realised stability | |  | |
| --- | --- | --- | --- | --- |
|  | *Low capacity* | *High capacity* | |  |
| Resilience | 0.06 | **0.08** |  | |
| Recovery | 0.55 | **0.83** |  | |
| Resistance | 0.25 | **0.57** |  | |
| Temporal stability | 11.78 | **19.62** |  | |

#### **Appendix 3**

### Summary statistics of empirical dataset

**Table S3: Empirical data on growth rates across species groups** (see file “compiled_growth_rates.xls”). Estimates refer to relative growth rates as defined by Hunt & Cornelissen (Hunt and Cornelissen 1997). The columns “org. estimate”, “org. unit”, and “org. measure” give the growth rate estimate, unit, and metric as published in the original studies. The column “conv. estimate” refers to the converted estimate, if applicable. References of original studies: (Ahn *et al.* 2013; Benke & Jacobi 1986; Calbet & Landry 2004; Houghton *et al.* 2013; Lankiewicz *et al.* 2016; Levang-Brilz & Biondini 2003; Liang & Uye 1997; McConville *et al.* 2016; Møhlenberg 1995; Nielsen & Sand-Jensen 1991; Piwosz *et al.* 2018; Ramírez & Pringle 2006; Reddy & Debusk 1984; Schmidt *et al.* 2019).


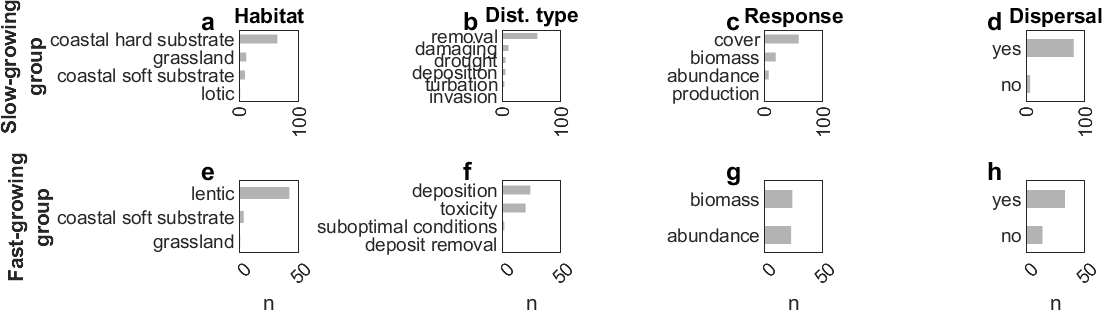


**Figure S2: Differences in studies of fast- and slow growing species groups.** Histograms show the number of observations per category for slower growing species (i.e. macrophytes and grasses; first row of panels) and faster growing species (i.e. microbes and phytoplankton; second row of panels). Studies of slow- and fast-growers differ in (**a,e**) studied habitat, (**b,f**) disturbance type, (**c,g**) response variable, and (**d,h**) whether experimental set-up is open to dispersal.
